# Optimizing Research Methods to Understand HIV-Exposed Uninfected Infant and Child Morbidity: Report of the Second HEU Infant and Child Workshop

**DOI:** 10.3389/fimmu.2016.00576

**Published:** 2016-12-06

**Authors:** Amy L. Slogrove, Moherndran Archary, Mark F. Cotton

**Affiliations:** ^1^Faculty of Medicine and Health Sciences, Department of Paediatrics and Child Health, Stellenbosch University, Stellenbosch, South Africa; ^2^Faculty of Health Sciences, Centre for Infectious Disease Epidemiology and Research, School of Public Health and Family Medicine, University of Cape Town, Cape Town, South Africa; ^3^Division of Paediatric Infectious Diseases, Department of Paediatrics, University of KwaZulu-Natal, Durban, South Africa; ^4^Faculty of Medicine and Health Sciences, Division of Paediatric Infectious Diseases and Family Clinical Research Unit (FAM-CRU), Department of Paediatrics and Child Health, Stellenbosch University, Stellenbosch, South Africa

**Keywords:** HIV-exposed uninfected, infant feeding, early infant diagnosis, antiretroviral drugs, *in utero*, Cohort Studies, surveillance systems, pharmacovigilance

The first HIV Exposed Uninfected (HEU) Infant and Child Workshop was held in Vancouver in July 2015, hosted by the Child and Family Research Institute at the British Columbia Children’s Hospital and University of British Columbia. This event brought together 50 clinicians, epidemiologists, and basic scientists to review current knowledge of HEU infants, their clinical course, immunologic differences, and risk for neurodevelopmental and infectious morbidity. This *Frontiers in Immunology* Research Topic, “Immune mechanisms underlying the increased morbidity and mortality of HIV-exposed uninfected (HEU) children,” is a product of the first HEU workshop synthesizing the evidence in the field. It was clear from the first workshop that there is a committed community of researchers who have identified the need to understand the mechanisms of increased morbidity and mortality in HEU infants and children, but evidence to intervene and mitigate these risks is lacking.

In high HIV burden countries, all infants and children, irrespective of HIV exposure, are vulnerable to high rates of infant and child mortality (1). In this context, the essential question is whether HEU children are any different than HIV-unexposed uninfected (HUU) children experiencing similar nutritional, environmental, and social constraints to health. To this end, particular research methodological principles require reinforcing in future HEU research. It was these methodological challenges and possible solutions that formed the theme of the second HEU Infant and Child Workshop attended by 75 HEU researchers and hosted by the KwaZulu-Natal Research Institute for Tuberculosis and HIV at the University of KwaZulu-Natal in Durban, South Africa. We report on the specific methodological challenges tackled during the workshop and steps to move forward.

## Accurate Classification of HIV-Exposed Children

Accurately excluding HIV infection and classifying infants as HEU is essential to ensuring integrity of HEU morbidity research to be certain that morbidity assigned to HEU infants is not in fact due to undiagnosed HIV-infection. Moreover, misclassification of HEU infants as HIV-infected or delayed diagnosis of HIV infection have detrimental life-long implications. The infant HIV diagnostic dilemmas emerging in the setting of expanding maternal antiretroviral therapy (ART), prolonged infant postnatal prophylaxis, and decreasing vertical HIV transmission were considered. With declining prevalence of infant HIV infection, standard HIV polymerase chain reaction (HIV-PCR) tests with unaltered sensitivity and specificity now have reduced positive predictive value. This makes confirmation of positive HIV-PCR results essential to rule out false positive tests. Prolonged infant postnatal antiretroviral (ARV) prophylaxis can reduce the viral load in an intrapartum HIV-infected infant sufficiently to result in a false negative HIV-PCR result at 6 weeks of age, requiring that diagnosis of infants as HIV-uninfected is reserved until all postnatal ARV prophylaxis has ceased (2). In the presence of maternal ART, extended persistence of maternal antibodies beyond 18 months of age has been observed in up to 14% of confirmed HEU infants requiring re-evaluation of the lower age limit at which a positive HIV serologic test is considered diagnostic of HIV (3). Additionally, there is a lack of precision in definitions related to HIV exposure with conflation of the terms HIV-exposed and HEU. Findings that have been described in original research as pertaining to HIV-exposed children, including both HIV-infected and HIV-uninfected, are cited in narrative reviews as relating to HEU children specifically (4–6).

*Moving forward: confirmation of positive HIV-PCR results should be the standard of care. Re-evaluation of the appropriate lower age limit at which a serologic HIV test can be used to diagnose HIV infection is needed. Timing of postnatal ARV prophylaxis and breastfeeding cessation needs to be taken into consideration when diagnosing infants as HIV-uninfected. The term HEU should be reserved for HIV-exposed children in whom HIV infection has been excluded. For interpretation of interim results while HEU infants are still breastfeeding and thus at risk of HIV infection, clear presentation of the timing of HIV testing in relation to ongoing HIV exposure is advised*.

## Understanding HEU Immune System Aberrations and Their Role in Infectious Morbidity

A critical look was taken at the evidence for immune differences in HEU compared to HUU children. With little evidence for quantitative or functional differences in major lymphocyte subsets, there is fairly strong evidence for increased immune activation in HEU compared to HUU infants (7–9). Drivers of immune activation in HEU infants are yet to be elucidated but may include exposure to HIV viral particles observed to stimulate a non-specific hypergammaglobulinemia persisting long after waning of maternally derived IgG, or in response to subclinical chorioamnionitis in HIV-infected women (10). Much evidence of immune activation in HEU children comes from the pre-ART *era* when the maternal *in utero* environment was substantially different to the environment in the ART era with HIV viral load suppression and improved maternal health. Although immune deficits that may explain the clinical pattern of HEU infectious morbidity have been hypothesized, no study has linked HEU immunological aberrations to clinical manifestations (6).

*Moving forward: adequately powered prospective studies are needed that evaluate immunologic differences between HEU and HUU children in conjunction with clinical outcomes in the era of universal maternal ART*.

## Understanding the Role of Additional Exposures in HEU Child Morbidity

HIV-exposed uninfected children experience exposures beyond HIV exposure that may play a role in mediating, modifying, or confounding the relationship between HIV exposure and the morbidity experienced. Two specific exposures considered at the workshop were the role of breastfeeding in reducing HEU morbidity and how best to measure this and how to evaluate the safety of *in utero* and postnatal ARV exposure.

### Suboptimal Breastfeeding

Many longitudinal studies that consider child health outcomes collect detailed infant feeding information over time. The hope in collecting rich feeding detail is that it will be possible to accurately reflect the infant feeding reality and its effect on the outcome of interest in statistical models. Nevertheless, infant feeding status is often reduced to a single variable of simplified categories at a single time point. Discussion of how to optimally measure and compare feeding exposures in HIV-exposed infants raised the tension between measurement for routine surveillance purposes, which requires pragmatism and focus on a few key questions, versus measurement for research purposes that may require more nuanced detail and sophisticated analytic techniques to understand the role of breastfeeding in reducing HEU infant morbidity. The lack of reliable information on rates of breastfeeding in HIV-infected women, critical information in accurately estimating postnatal HIV transmission rates, was highlighted. Qualitative work was shared that showed infant feeding choices to be ongoing for mothers despite making an initial decision and that health-care providers who are highly influential in these initial choices often lack the skills to adequately transfer messages to HIV-infected mothers about the risk–benefit ratio of infant feeding choices. It is clear that breastfeeding is beneficial to child health irrespective of HIV exposure and every effort must be made to support both HIV-infected and HIV-uninfected mothers to successfully and safely breastfeed. However, recent evidence indicates that even breastfed HEU infants experience greater infectious morbidity than HUU infants (11, 12).

*Moving forward: pragmatic indicators of breastfeeding are needed in national and global monitoring systems to accurately estimate the risk of postnatal HIV transmission. Interventions to support successful and safe breastfeeding need further investigation. Mechanisms of excess infectious morbidity in breastfed HEU infants, e.g., differences in breast milk quality, household pathogen exposure, and immune susceptibility amongst others, need to be understood*.

### *In Utero* and Postnatal Antiretroviral Exposure

Access to maternal ART has made vertical HIV infection a preventable disease, and we must continue to strive for its elimination. There is also the imperative though to ensure that the safest vertical transmission prevention interventions available are provided. Little has been done to set up pharmacovigilance systems in high HIV burden countries. Most ARVs currently used are considered non-teratogenic; however, short-, medium-, and long-term effects of ARV exposure beyond congenital anomalies are not well understood. Findings from Botswana shared at the workshop indicate that compared to HEU infants with *in utero* exposure to zidovudine monotherapy, HEU infants with triple ARV *in utero* exposure had significantly lower length-for-age at 24 months, even after adjusting for maternal disease severity (13). This is just one example of recent findings highlighting the urgent need for large scale pharmacovigilance and ARV safety surveillance that can accommodate the pace of expanding universal ART and inevitably changing regimens. The workshop heard about encouraging advances being made in statistical methods to appropriately evaluate the safety of *in utero* exposure to individual ARVs in the context of changing ART regimens over time, multiple concurrent ARV drug exposures and confounding by indication (14).

*Moving forward: establishment of pharmacovigilance surveillance systems and collaboration and pooling of research data is going to be necessary to achieve sufficient numbers of ART-exposed infants to confidently and continuously establish safety as vertical transmission prevention interventions evolve*.

## Study Design Strategies

Evidence of elevated HEU morbidity and mortality has largely come from secondary analyses of vertical transmission prevention clinical trials, with few studies primarily designed to compare HEU and HUU infants for a specific morbidity (15). Despite the large size of the HEU child population in high burden countries (e.g., up to 30% of children in South Africa), recent studies have lacked power to draw definitive conclusions about differences between HEU and HUU children (12, 16). Indirect comparisons of HEU children to general child population rates have provided useful information in the past but are no longer helpful to understand how HEU and HUU children from similar communities compare (17–19). To understand mechanisms of vulnerability in HEU children, adequately sized cohort studies primarily comparing HEU and appropriate HUU control children are required. For the evaluation of long-term ARV exposure safety though, establishing consented cohorts of HEU children with direct follow-up was not found to be feasible in the United Kingdom (20). As an alternative, routine national data is being used to monitor death and cancer in children born to HIV-infected mothers and exposed to ARVs. Efforts in the Western Cape Province of South Africa are leveraging the province-wide patient unique identifiers to establish a cohort of all pregnancies that can link mother–baby pairs to digitally available exposures including pharmacy records for ARV and other drugs and laboratory records for HIV diagnostics. This will allow for evaluation of birth outcomes in relation to ARV *in utero* exposure, hospitalizations for infectious and other events and future association with rare events such as malignancies. Harmonization of outcome measures, particularly birth outcomes, infectious morbidity, growth, and neurodevelopment measures could aid HEU researchers in more rapidly resolving outstanding questions through better comparability of studies across different settings and different study designs.

*Moving forward: adequately sized cohort studies are necessary to determine mechanisms of HEU vulnerability in early childhood. Surveillance strategies using existing population-based demographic surveillance sites and national databases may be a more feasible strategy though than cohort studies for long-term evaluation of in utero ARV exposure safety. Both cohort and surveillance studies need to pay attention to potential confounding by socioeconomic circumstances and infant feeding differences*.

## Conclusion

HIV-exposed uninfected infants and children are currently not identified within global HIV monitoring frameworks, such as the UNAIDS Global AIDS Response Progress Reporting system. Consequently, there is little focus within countries on measuring outcomes in HEU children beyond prevention of vertical HIV infection. While the bigger picture of improving health and well-being for all children must be kept in mind, and one group should not be prioritized to the detriment of the other, the evidence is mounting that HEU children experience excess morbidity and mortality (21, 22). There is a pressing need for clinicians, program implementers, researchers, policy makers, and HIV-affected families to form an organized community of HEU child advocates that can coordinate placing HEU children in a more prominent position on the global HIV research and monitoring agenda. The workshop concluded with a reminder to think more broadly about vertical transmission prevention aims and to re-envisage Preventing Mother to Child Transmission rather as Promoting health of Mothers and Their Children Together with supporting Fathers and Families (PMTCT F^2^). We look forward to the third workshop in Paris in July 2017.

## Author Contributions

ALS conceived of and drafted the manuscript. MA and MFC gave critical intellectual input and reviewed and approved of the manuscript.

## Conflict of Interest Statement

The authors declare that the research was conducted in the absence of any commercial or financial relationships that could be construed as a potential conflict of interest.
